# A Squib

**Published:** 1859-08

**Authors:** 


### A SQUIB.

We are not an editor, but a dentist. We are also an individual, but we write ourself *we* because we have not yet attained our dental majority, and must, therefore, rest conented in our plurality. We hope. *Now*, we want to say something about "fang-filling," as it is termed, and in order that our conclusions may be well founded, we will first reconnoiter our premises. Ah! here they are, interrogatively stated in "the Register" for July—we copy. "When the nerves of teeth are destroyed, is it proper to fill the fang and crown, or the crown only?" Since we first saw that paragraph in print we have taken frequent opportunities for viewing it.—We like to look at it. We gaze and wonder. We wish to know the writer. Did a man write it? Who lived in 1853? What was then fashionable? "See how they run." Take care reader! But isn't it fascinating, eh? Is'nt it bewitching? Just to think that *sometimes*, in a very *difficult case,* say when the 'fang' is in the vicinity of the stomach, head-waters threatening to inundate the canal, the patient impatient, and one very tired, that then, in such case, *it may be* "proper to fill the crown only." Goodness! what a glorious possibility. There *is* then a doubt! Gentlemen of the jury, you will give yourselves the benefit of the doubt on the dicta of the crown judge, and bring the assembly to its "extreme end" by singing "There is no more work," after which the Doc's elegy!

We do most heartily wish that we had never seen this sentence in question, it so completely obtains us. We were getting along so steadily, and contentedly in the warm sunlight of reason, reflected from common sense, that we had wholly ignored the shady side of this matter, and should probably have remained in thoughtless ignorance until doomsday, had not this sermon on the "go it blind" system chanced to have met our eye. We don't yet understand the thing, don't believe that we can; but for that very reason think there must be something in it—mystery is so enchanting!

*Do*, somebody, come to our aid or we shall perish before the sphinx. How *can* it be "proper to fill the crown only"?—Why not fill the mouth permanently with gutta percha after thoroughly drenching the patient, and then trust to the Dame to carry on the economy, by endosmotic absorption? Is there not poetry in the idea of abhorrent nature embracing her old-time enemy, the vacuum, thus symbolizing the perfection of the W-hole? Sure the Millenium is at hand!

Seriously, at last, let us take a *simple demonstrable* view of the subject of filling teeth, the pulps of which have been destroyed.

A central incisor is carious and the pulp-cavity exposed. If the inclosed tissue still retains its vitality, an escharotic may be applied, which will destroy its sensibility, and reduce it to the level of inorganic matter. If the pulp shall have long ago died a natural death, and alveolar abscess have supervened, then the cavity will be found to be filled with inorganic matter as in the first case supposed. Now, what has a dentist to do? It seems to us that his ordinary experience joined with a little of the leaven, common-sense, *must* lead him to take out the corruptible and put in the incorruptible; to remove the carious dentine and the deadened pulp, and *fill the cavity from apex to base* with gold or its equivalent.

And the second is like unto the first, differing only in the preparatory treatment. The abscess is to be treated topically until there is no longer any discharge into the pulp-cavity, or through the process, when the tooth is to be filled as before.

We think this a plain statement of a plain case, embodying the whole subject and capable of practical demonstration in the manner above described; embracing cause, effect, and cure of the diseased condition. With theories, gaseous or galvanic, we at present have nothing to do.

We shall take occasion to refer again to this subject and treat it more in detail, since its importance seems abundantly to justify the attempt. W. S. H.

